# Supplementary material for: A cross-national study examining imaginary companions and face pareidolia in British and Chinese adults
Source: PLoS One. 2026 Jan 23;21(1):e0325581. doi: 10.1371/journal.pone.0325581 (PMC12829835; doi:10.1371/journal.pone.0325581)
Supplement: S1 File — (DOCX) [file pone.0325581.s001.docx]

## *Analysis of Interrelations*

### Relations Between Pareidolia and IC perceptual experiences and Imagination

A bivariate correlation was run to determine whether pareidolia hits and false alarms related to the imagination questions and reports of sensory experiences with ICs. Hearing one’s IC was positively related to the amount of pareidolia hits *r =* .280, While other sensory experiences were positively related to pareidolia false alarms *r =* .223 and so was how creative participants rated themselves in their jobs *r* = .146. Pareidolia hits and false alarms were significantly positively related as well *r =* .273. For more inter relations between these variables, see table 1.

## Table 1. Inter relations between variables

| Variable | *n* | 1 | 2 | 3 | 4 | 5 | 6 | 7 |
| --- | --- | --- | --- | --- | --- | --- | --- | --- |
| 1. Can you see your IC | 142 | __ |  |  |  |  |  |  |
| 2. Can you hear your IC | 142 | .472 | __ |  |  |  |  |  |
| 3. Other sensory experiences with IC? | 142 | .344** | .190* | __ |  |  |  |  |
| 4. Total pareidolia hits | 291 | -.019 | .280** | .154 | __ |  |  |  |
| 5.Total pareidolia false alarms | 291 | -.007 | .143 | .223** | .273** | __ |  |  |
| 6.How Creative are you in your job? | 291 | .165* | .005 | .117 | .176 | .146* | __ |  |
| 7.How imaginative are you? | 291 | .195* | .004 | .143 | .099 | .016 | .384** | __ |

**. Correlation is significant at the 0.01 level (2-tailed).

*. Correlation is significant at the 0.05 level (2-tailed).

### Relations Between Pareidolia and IC perceptual experiences Imagination and Culture

We wanted to look at these same variables for each country to account for any differences in associations. First, we looked at only the participants in China. In this group, hearing one’s IC no longer related to the amount of pareidolia hits, but instead other sensory experiences became significant and was associated with pareidolia hits *r =* .261. Other sensory experiences remained a predictor of pareidolia false alarms as well *r =* .291. The two pareidolia scores were also associated with each other *r = .*335. For more inter relations between the variables in the Chinese group see table 2.

Table 2. Interrelations between variables: China

| Variable | *n* | 1 | 2 | 3 | 4 | 5 | 6 | 7 |
| --- | --- | --- | --- | --- | --- | --- | --- | --- |
| 1. Can you see your IC | 77 | __ |  |  |  |  |  |  |
| 2. Can you hear your IC | 77 | -.106 | __ |  |  |  |  |  |
| 3. Other sensory experiences with IC? | 77 | .293** | .148 | __ |  |  |  |  |
| 4. Total pareidolia hits | 185 | .029 | .220 | .261* | __ |  |  |  |
| 5.Total pareidolia false alarms | 185 | -.141 | .163 | .291* | .335** | __ |  |  |
| 6.How Creative are you in your job? | 185 | .208 | .169 | .241* | .118 | .120 | __ |  |
| 7.How imaginative are you? | 185 | .227* | -.064 | -.005 | .042 | .015 | .448** | __ |

**. Correlation is significant at the 0.01 level (2-tailed).

*. Correlation is significant at the 0.05 level (2-tailed).

Next, we looked at the participants in the UK group to determine if there were differences in associations from the combined groups. In the UK, associations with hearing one’s IC was positively related to the amount of pareidolia hits *r = .*373. No other significant associations were found between pareidolia and the perceptual experiences around one’s IC. For more inter relations between the British group see table 3.

## *Table 3. Interrelations between variables: UK*

| Variable | *n* | 1 | 2 | 3 | 4 | 5 | 6 | 7 |
| --- | --- | --- | --- | --- | --- | --- | --- | --- |
| 1. Can you see your IC | 65 | __ |  |  |  |  |  |  |
| 2. Can you hear your IC | 65 | .314* | __ |  |  |  |  |  |
| 3. Other sensory experiences with IC? | 65 | .433** | .234 | __ |  |  |  |  |
| 4. Total pareidolia hits | 106 | -.094 | .373** | -.043 | __ |  |  |  |
| 5.Total pareidolia false alarms | 106 | .187 | .156 | .137 | .156 | __ |  |  |
| 6.How Creative are you in your job? | 106 | .114 | -.132 | -.012 | -.003 | .198* | __ |  |
| 7.How imaginative are you? | 106 | .172 | .060 | .412** | .238* | .096 | .295** | __ |

**. Correlation is significant at the 0.01 level (2-tailed).

*. Correlation is significant at the 0.05 level (2-tailed).

*Analysis of Age*

Because there were no specific ages but rather age ranges, we chose to remove the analyses from the main manuscript. We acknowledge this is a limitation to the research. Age groups were analysed finding no age effects *χ*^2^ (1, *N* = 181) = 1.06, *p* = 0.303 without the 50 and over group. The chi square with the over 50 group was also non-significant, but 2 cells were less than 5.

*ICs, Imagination and Country Status*

We then analysed the participant’s reports of their imaginativeness in reference to their IC and country status. We found significantly higher self-report scores of imagination in both those with current *t* (1,281) = 3.67, *p* <.001 and past *t* (1,283) = 3.08, *p* = .001 ICs. However, this did not remain true for participant report of creativity within their jobs for those with either current *t* (1,281) = 0.19, *p* = .426 or past *t* (1,283) = 0.48, *p* = .316 ICs.

Country differences were found in self-report of imaginativeness, where individuals from the UK reported significantly higher imaginativeness *t* (1,289) = -2.33, *p* = .010. Again, when asked how creative one is currently in their job, the difference disappeared *t* (1,289) = 0.32, *p* = .376.

*Development of Pareidolia Task*

The presence or absence of a face pareidolia in the images presented to participants was established in a pilot study. In this pilot, each image from the final task was presented to a group of seven participants (who did not take part in the full study). Images were presented using Microsoft PowerPoint, in a fixed, random order. Participants were given unlimited time to detect whether or not a face pareidolia was present or absent in each image. All participants detected the face pareidolia in the 24 images that we have classed as “pareidolia present”. None of the participants detected a face pareidolia in the12 images that we have classed as “pareidolia absent”.

*Analyses of Pareidolia Task Performance Using Signal Detection Parameters*

In addition to examining group differences in performance on the pareidolia task using participant’s ‘raw’ scores, we examined group differences using signal detection parameters. We calculated *d'* as a measure of sensitivity. This was found by subtracting the standardized false-alarm rate from the standardized hit rate. We calculated C-score as a measure of response bias. This was found by multiplying the sum of the standardized false-alarm rate and the standardized hit rate by -0.50.

Both C-score and *d'* were not normally distributed. We therefore used non-parametric tests to examine group differences on these outcomes. We tested whether the differences between the IC Groups were significant using a pair of Mann-Whitney U tests. The difference in C-score between the Group without a Childhood IC (median = 0.53, IQR = 0.54) and the Group with a Childhood IC (median = 0.56, IQR = 0.46) was not significant, U = 9822, z = 0.21, p = .83, r = .01, two-tailed test. Similarly, the difference in *d’* (i.e., sensitivity) between the Group without an IC (median = 1.89, IQR = 1.90) and the Group with an IC (median = 1.94, IQR = 1.00) was not significant, U = 8996.50, z = 1.41, p = .16, r = .08, two-tailed test.

The difference in C-score between the Group without a Current IC (median = 0.54, IQR = 0.50) and the Group with a Current IC (median = 0.46, IQR = 0.68) was significant, U = 3446, z = 2.20, p = .028, r = .13, two-tailed test. In contrast, the difference in *d’* between the Group without a Current IC (median = 1.93, IQR = 0.90) and the Group with a Current IC (median = 2.28, IQR = 0.90) was not significant, U = 3132, z = 1.77, p = .08, r = .10, two-tailed test.
