# Supplementary material for: A cross-national study examining imaginary companions and face pareidolia in British and Chinese adults
Source: PLoS One. 2026 Jan 23;21(1):e0325581. doi: 10.1371/journal.pone.0325581 (PMC12829835; doi:10.1371/journal.pone.0325581)
Supplement: Attachment — Submitted filename: Plosone IC Pareidolia_resub01_response_v1_ds_ediyts cf.docx [file pone.0325581.s005.docx]

August 23^rd^ September, 2025

Dear Editors,

Thank you for your review of the manuscript entitled*,* *A Cross-National Study Examining Imaginary Companions and Face Pareidolia in British and Chinese Adults*. We have amended the manuscript according to the feedback and have laid out our changes below. The main text is now 6291 words excluding the abstract and tables/figures. It is now 30 pages in length including the main unblinded text, references, tables and figures. It contains 4 tables 2 figures and appendices.

Sincerely,

Paige E. Davis, PhD, FHEA,

Lecturer in Psychology

University of Leeds

School of Psychology

LS2 9JT

+44 (0)7542 098888

p.e.davis@leeds.ac.uk

**Reviewer #1**

| 1. The conclusion of the abstract comes across as too broad and somewhat disconnected from the actual study scope. For example, the sentence: “Taken together, the results provide more information around the role of top-down processing in both imaginary companion creation and hallucination. This could be a way to learn about the developmental trajectory of hallucination from non-pathological to pathological.” seems overly speculative. Since the study is mainly of exploratory nature and focuses on ICs and face pareidolia, it does not directly investigate hallucinations, especially not pathological ones. For instance, Romagnano et al. (2022) examined face pareidolia in individuals with schizophrenia and found no significant differences in pareidolia perception between patients with or without hallucinations. | Thank you for this suggestion. We have now changed the conclusion to reflect what we found rather than going too broad with the implications. This is what we have:  *Taken together, the results provide more information around imaginary companion creation in China and the UK as well as the role top-down processing may play in imaginary companion interactions.* |
| --- | --- |
| 2. Participants: It would be helpful to clarify whether participants were screened in the questionnaire for psychiatric, developmental, or neurological conditions, given the significant impact such conditions can have on social cognition and related perceptual processes. | We have now added a sentence in the method stating:  *Participants were not screened for any psychological, developmental or neurological conditions.*  (lines 181-182)  As well as a sentence in the limitations using the wording suggested in this review. Thank you.  *Another consideration is that participants were not screened for any psychiatric, developmental or neurological conditions and given the significant impact on social cognition and perceptual processes, having this information could improve our ability to evaluate the results.* (lines 437-439) |
| 3. Visualization: The paper would benefit from improved visualization. Including examples of the stimuli used and/or graphical representations of key results would help readers better understand and interpret the findings. | We have now added a figure which shows the stimuli.  We have also added a figure which is a graphical representation of the pareidolia hits. We think this improves understandability of the findings. |
| 4. Discussion: The authors mention that they were surprised to find cultural differences between the UK and China in face pareidolia, referencing a study that found no significant differences between two European subcultures (reference 33). However, given the well-documented differences in face processing between Western and East Asian populations, differences in pareidolia perception might indeed be expected. This could be discussed in more depth, potentially referencing additional literature.  Additionally, Zhou and Meng (2021) analyzed face pareidolia using signal detection theory (SDT). Incorporating similar analyses, such as sensitivity (d') and decision criterion (c), could provide more insight into the nature of the cultural differences observed and should be considered either in this paper or in future work. | Thank you for bringing this issue up. After re-reading the paper about cultural differences we took the decision to refine the argument in the introduction and explain that the study (original manuscript reference 33) is in two European subcultures rather than Eastern and Western cultures. We also explained further about the large individual differences found in pareidolia. This set the argument up in the discussion to focus more on differences in face processing.  *Because pareidolia tasks ask participants to view pictures and make assessments of objects resembling faces with no clear race or ethnicity, rather than real faces, these tasks have been used in cross cultural studies and Romagnano and colleagues have even attempted to rule out own-culture and gender biases when participants look at faces (31). However, Romagnano’s research examined two European cultures which were situated closely together. There have not been any studies to the authors knowledge on individual differences in pareidolia in Chinese and British Adults. Large individual differences in the experience of face pareidolia has been reported in many studies over domains like gender, developmental period, personality and neural mechanism (32,33).* (line 145-153)  We also changed the discussion to explain that face processing differs with culture and made your point about this transferring to pareidolia. See below paragraph for the change:  *When considering the pareidolia scores, there were cross-national differences in false alarms where Chinese participants made significantly more false alarms, but there were no differences in pareidolia hits. It is well documented that there are cultural differences in face processing (e.g.46). For example, in Europe, French-speaking Swiss participants and German participants had different patterns of face tuning (47). More diverse culture shifts comparing Canadian and Chinese participants show that even the way visual information about a face is received can be impacted by culture (48). It may follow that, with these cultural differences in face processing, there may also be differences in pareidolia perception.* (lines 384-389)  We added Zhou and Meng (2020) and Wang and Yang (2025) to intro and Caldara (2017), Pavlova et al (2018) and Tardif et al (2017) into the discussion.  Thank you for the suggestion to include analyses involving signal detection parameters. We have calculated these values and have included analysis employing these parameters in the Supplementary Materials. Note that the pattern of effects when using d' and C are consistent with the main analyses. Group differences were not significant when we compared participants with versus without a childhood IC. Similarly, the group difference in d' was not significant when we compared participants with versus without a current IC. However, there was a significant group difference in C when we compared participants with versus without a current IC. Consistent with the greater number of ‘hits’ made by participants with a current IC, they also had a lower mean C-score (i.e., they had a more liberal bias, towards responding that a face was present in the images). |

**Reviewer #2-We appreciate that no AI tools were used and thank you for taking the time to do this yourself.**

| 1. Overall Changes: I am struggling to understand the use of face pareidolia as a proxy for hallucinations, particularly given the task that was used to measure face pareidolia in this study. Hallucinations, to the best of my understanding, are hearing, seeing or experiencing things for which there is no basis of reality, or for which there is no objective evidence of existence. With hallucinations, what is “real” or “true” appears to be a key consideration in measuring what is and is not a hallucination (at least in western cultures). However, seeing faces in visual stimuli such as abstract images, clouds, objects, etc. is a subjective experience in which a basis of reality may not matter as much, and to the best of my knowledge, more closely associated with individual differences in anthropomorphism in the literature. With abstract imagery, the presence of a face in visual stimuli could simply be “in the eyes of the beholder.” In other words, seeing a face in abstract visual imagery is neither “true” nor “false”; it just is.  And that brings me to my concern about the task designed to measure face pareidolia, where “correct hits” are described as suggestive of a greater propensity for hallucinations, and “false positives” are discussed simply as errors. If the task was designed as a measure or proxy for the experience of hallucinations, then the “false positives” are of greater interest than the “correct hits.” And in this study, there were no differences in false positives based on IC status (past or current, absent or present), which would change the authors’ interpretation of the findings. However, if the task is not intended to serve a proxy for hallucinations but rather a direct measure of face pareidolia alone, then it is unclear why the measure needed to have “correct” or “incorrect” responses. In other words, even with the same task, why not simply measure for each participant the number of images in which a face was detected? | Apologies that this was not clearer in the original manuscript. The pareidolia task is not meant to measure/be a proxy for experiencing hallucinations. Instead, the task aims to measure ‘top-down processing’ (i.e., the extent to which perception is shaped by expectations) – a process that we think plays a role in the development of both ICs and hallucinations. We have tried to make this clearer by editing a sentence in the Method section:  *The face pareidolia task designed by Smailes and colleagues was used to measure top-down processing presented to the participants through the Gorilla platform*. (lines 228-230)  The reviewer is right that, to some extent, face pareidolia are ‘in the eye of the beholder’. However, to make this task less ambiguous we developed it in the following way, so that we think it is appropriate to consider some responses to be ‘correct’ and some to be ‘incorrect’: The presence or absence of a face pareidolia in the images presented to participants was established in a pilot study. In this pilot, each image from the final task was presented to a group of seven participants (who did not take part in the full study). Images were presented using Microsoft PowerPoint, in a fixed, random order. Participants were given unlimited time to detect whether or not a face pareidolia was present or absent in each image. All participants detected the face pareidolia in the 24 images that we have classed as “pareidolia present”. None of the participants detected a face pareidolia in the12 images that we have classed as “pareidolia absent”. We have added this information about how the task was developed into the Supplementary Materials.  RE: The point about ‘pooling’ participants ‘face-present’ responses (i.e., summing ‘hits’ and ‘false alarms’). To some extent, the additional analyses we have performed (and included in the Supplementary Materials) using C-score as an outcome variable achieves this (as C-score is calculated using both hits and false alarms). We have, however, left this analysis as supplementary and have focused on the ‘raw’ hits and false alarms because we believe that hits and false alarms measure different processes. That is, we think that individual differences in top-down processing are measured by the number of hits a participant makes. However, we do not think that individual differences in top-down processing are measured by the number of false alarms a participant makes. Consistent with this claim, the correlation between the number of hits and number of false alarms a participant makes was medium-sized in this study (rho = .326) and was close-to-zero in a previous study that one of us (DS) conducted (rho = .07; see [these](https://www.tandfonline.com/doi/suppl/10.1080/13546805.2019.1700789?scroll=top) supplementary materials). If both number of hits and number of false alarms both measured top-down processing, we would expect these correlations to be stronger. |
| --- | --- |
| 2. Suggestions: In the literature review, make a stronger case as to how seeing faces in visual stimuli has links to hallucinations and to ICs, from a theoretical perceptive and in terms of measurement. Reconsider the description of the measure of face pareidolia (i.e., if it is indeed being used as a proxy for measuring hallucinations or if is not meant to imply anything beyond face pareidolia). If it is being used as a proxy for measuring hallucinations, please explain why “false positives” were not the primary variable of interest. | We have edited the Introduction so that it is hopefully clearer that our logic is as follows: 1 = ICs and hallucinations/hallucinatory experiences (H/HEs) are similar in some ways; 2 = They may be similar in that the same processes may play a causal role in the development of both ICs and H/HEs; 3 = Past research has shown that hearing words in ambiguous speech sounds (which probably reflects elevated top-down processing in the auditory domain) is associated with both ICs and H/HEs; 4 = Visual H/HEs are associated with a tendency to detect more face pareidolia (which probably reflects elevated top-down processing in the visual domain); 5 = Given the ICs often have a visual component, it seems plausible that having an IC will be associated with elevated top-down processing in the visual domain; 6 = We, therefore, expected that participants with an IC would detect more face pareidolia in the task than would participants without an IC.  RE: the comment about why false alarms were not our primary outcome measure – please see our response above. |
| 3. Literature Review Again, in the literature review, I am struggling to understand how top-down processing, ICs, hallucinations, and face pareidolia are all theoretically connected. Consider adding a schematic image to clarify for the reader. | Thank you for this idea, we have edited the Introduction so that it is hopefully clearer how we think these factors are related. |
| 4. The literature review also assumes prior knowledge of other ideas/constructs, such as theory of mind and top-down processing. Provide brief definitions so that readers who are not as familiar with this literature can understand the points that are being made. | Thank you for this comment. The ideas of theory of mind and top-down processing now have been explained in the introduction.  *This elevated performance in tasks around theory of mind, or the understanding that others can take different perspectives, as well as having different thoughts feelings or ideas, has been replicated in China.* (lines 105-107)  *that a person’s tendency to detect speech sounds in ambiguous auditory stimuli reflect individual differences in auditory top-down processing (processing using prior knowledge or expectations to interpret ambiguous stimuli)* (lines 123-125) |
| 5. In lines 98 – 102, it would be helpful to understand the interplay between cross-cultural differences in face pareidolia and ICs. Perhaps speculate as to what this interaction might be or look like in this section. In addition, more information about how face pareidolia tasks have been used to assess cultural and gender biases would be helpful. | Please see 2^nd^ comment of your review. We are hoping that this is now clear.  We have now added in the task used to look at pareidolia and culture in the Romagnano and colleagues paper.  *Because pareidolia tasks ask participants to view pictures and make assessments of objects resembling faces with no clear race or ethnicity, rather than real faces, these tasks have been used in cross cultural studies and Romagnano and colleagues have even attempted to rule out own-culture and gender biases when participants look at faces (31). However, Romagnano’s research examined two European cultures which were situated closely together. There have not been any studies to the authors knowledge on individual differences in pareidolia in Chinese and British Adults. Large individual differences in the experience of face pareidolia has been reported in many studies over domains like gender, developmental period, personality and neural mechanism (32,33).* (line 145-152) |
| 6. Also, in the literature review, justify why children were not included in this study or the focus of the study, as retrospective reports of ICs are problematic due to memory/recall, and research question 1 (as it is written) suggest that adult and child reports of ICs will be compared. | Thank you for this piece of feedback. We have now changed the first research question to be clearer stating:  *Would there be significant cross-national differences between adult report of IC creation in childhood and adulthood between UK and Chinese participants?* (lines 158-159)  We chose to look at adults as the study was investigatory in nature and we would be able to gather the data online rather than by going into an environment to gather data with children. Using adult report of ICs is standard practice in many studies. We are currently gathering data around children, however only in one culture. We’ve added an additional rationale for this with the aims:  *The aims of this study were to examine relationships between cross-national status, imaginary companion creation and face pareidolia in adulthood. Children were not included because of the investigatory nature of the study and the method of delivery being an internet platform.* (lines 154-157) |
| 7. Finally, throughout the manuscript, please take care in discussing findings using developmental language, as this was a study of adults and did not make direct comparisons with children, or follow participant development over a period of time (see lines 322 – 323 as an example). | We have now gone through and taken out the paragraph in the introduction which uses developmental language. We have also amended the claim on (original line 322-323) to both tone it down and suggest future research.  *However, the finding that performance on the pareidolia task was associated only with the presence of a current, and not a childhood IC suggests that this could be a dynamic, state-like association rather than something that is highly stable as a person ages. More research would need to be done to support this theory, as we do not have longitudinal data and only retrospective accounts. (lines* 405-409) |
| 8. Method and Materials In lines 146-148, please clarify if participants completed both the face pareidolia task and the animacy task in randomized order, or whether they were randomly assigned to complete only one of these tasks. If the latter in the case, then clarify the number of participants in the whole sample and those that completed the face pareidolia task that were used in the analyses. | We have changed the method to clarify that both tasks were completed by participants in a random order.  *After the questionnaire a randomizer was used to direct the participant either to the face pareidolia task or another task focused on animacy which is not reported in this study. Once participants finished both face pareidolia and animacy tasks they were thanked for their time. All participants completed both tasks. Task order was randomized.* (lines 200-204) |
| 9. In lines 150 – 152, clarify if any ethics committees in China were involved in approving this study. | We have clarified that there are no affiliated Chinese institutions that were involved in this study on line 207. |
| 10. In line 178, please report timing in seconds rather than milliseconds for better reader comprehension. | We have changed this to 0.75 seconds on line 236. |
| 11. In lines 191 – 192, thank you for providing a link to the task. However, one figure showing a “correct hit” and a “false positive” would be helpful. | We’ve now added a figure with examples of the trials and response screen and a note which explains the scoring for each type of trial. |
| 12. Results In lines 242 – 244, provide a brief description or example of what is meant by “sensory experiences” of an IC. Also, note that as a reviewer, I did not have access to the supplementary materials (to the best of my knowledge) and would be interested in seeing them. | This has now been explained briefly:  *(e.g. whether the participant reported seeing or hearing their IC).* (line 315)  This has also been reported by another reviewer. We have appended it to the end of this response and will re-submit the materials again with this revision. |
| 13. Discussion In lines 286 – 288, I am concerned that this sentence reads as a biased and western take on what creativity is and how it manifests across cultures. No doubt that China has produced some of the most celebrated and venerated narratives, music, visual art, dance, film, food and fashion in recorded human history. | Thank you for this comment as we worked hard to try to avoid things that could be taken as western centric. We’ve now changed the sentences to be less value laden and concentrate on IC creation rather than having a creativity/imagination orientation.  *One possible explanation for this difference could be that British culture has been traditionally considered to be individualistic, valuing independence, novelty, autonomy and freedom, all values which may lead to creation of an imagined social relationship (Barth & and Stadtmann, 2024). Collectivist cultures like China have traditionally been more likely to value harmony, conformity and consensus, which may result in less individual ICs (Barth & and Stadtmann, 2024)* (lines 356-360) |
| 14. In lines 289 – 292, please discuss with more clarity how recruitment methods may have inflated the number of past and current ICs reported, and how this limits study findings without replication. Please also be sure to mention the use of retrospective reporting of ICs as a limitation. | There is now a discussion on how the recruitment may have gleaned more adults with ICs.  *Recruitment taglines stated participants needed. There was no mention of ICs in the recruitment, however the body of the advertisement said that the researchers were investigating imagination and face perception which may have steered those more interested in imagination. Although it has been found to be a robust platform, using prolific for recruitment in the UK may have gleaned a different group of respondents than pure social media recruitment as Prolific participants are paid a small amount for their participation, whereas in China there was simply a prize draw for a possible gift card.* (lines 367-373)  Attention is now paid to the retrospective IC report as well.  *Furthermore, adults retrospective reports of ICs could also pose a problem as memory of past ICs fades even as early as adolescence (54). Having children experiencing ICs while participating would help to determine if there are similar results.* (lines 431-433). |
| 15. There are a few minor typos throughout the manuscript. | We have found the more major one in the running head and have gone through to look for others. Thank you for this comment. |
| 16. In Table 1, please report the ns in raw numbers as well as in percentages. | Currently in table 1 the raw numbers of participants are reported followed by the percentage in brackets. |

**Reviewer #3**

| 1. The researchers discuss the research question in relation to hallucinations, however, I believe that the link between hallucinations and ICs (and pareidolia) needs to be explained/rationalized better in the introduction. The rationale behind the link between top-down processing and ICs are clear but I do find the link to hallucinations a little unclear. | Thank you for this comment, we have addressed this in the response to reviewer #2 comment #2. Please see above. |
| --- | --- |
| 2. It’s reported that “individual differences in the ability to detect face pareidolia have been found to correlate with the frequency of visual hallucination-like experiences in non clinical participants”. Here, I am confused as to what ‘visual hallucination-like experiences’ would be like. Can they also be considered signs of a person’s high creativity/imagination? How is the distinction made between creativity/imagination and ‘hallucination-like experiences’? | Thank you for bringing this to our attention. In the Introduction we have included examples of the types of hallucinatory experiences we are referring to. On line 75 we note that auditory hallucinatory experiences are often things like hearing a telephone ring when it has not. On lines 80 we note that visual hallucinatory experiences are often things like seeing a person/animal in one’s peripheral vision, when there is no person/animal there.  Where hallucinatory experiences ‘begin’ and imaginative/creative experiences ‘end’ is a very complex question, that is probably beyond the scope of this response. In short, it could be argued that internal experiences that are the product of imagination/creativity could be the ‘raw material’ from which hallucinations are formed. |
| 3. There were 12 questions regarding the ICs. I wonder how the answers to these questions were utilized. Did the researchers only use the questions asking whether whether the participant had a past or current IC, or did they also take into account participants’ answers to the other questions about sensory experiences, etc. when deciding whether what the participant is reporting a real IC or something else. This might especially be an issue when participants are reporting Personified Objects. Because, unless the answers to the other questions are taken into account, it is difficult to know whether the participants are reporting a real IC (PO) or whether they are simply thinking about their comfort and/or transition objects. | Some of the 12 questions (8 quantitative) were used in the supplementary material and analyses. They can be found at the bottom of this revision. Other (4 qualitative) responses were used to give examples of different ICs that were described in the study.  There is no documented way to methodically check if the researcher deems the IC a “true” IC through their follow up questions, however we trust that the adults answered truthfully. The established convention in the literature is to ask adults about their memory and experiences.  In past studies it has been the convention to define an IC and then ask the questions, so adults should have a clear idea of what it means when they are answering. We have added in the method section the definition we used. The new sentence explains:  *The first section asked their gender and age. Following these demographics, there was a definition of an IC according to the study’s parameters which read: an imaginary friend can be completely invisible or a doll or a toy which a child or adult has given a personality to and played with for at least 3 months. After the definition was read there were 12 questions about ICs.* (lines 213-217)  Taylor and Aguiar (2024) have mentioned this in their writing about identifying ICs and assert that although there are inherent issues in asking adults, the insight gained from maturity can produce deep descriptions and speculations about the behaviour. |
| 4. On the same questionnaire, participants were asked to rate themselves on creativity. Were the answers to these questions used in any analyses? It might be relevant and interesting, as the researchers discuss that “Collectivist cultures like China have traditionally been more likely to value harmony, conformity and consensus, which may be less creativity oriented” (p.13). | Thank you for this idea. As a result of this helpful comment, we have now used a t-test to determine that Adults in the UK rate their imaginativeness as significantly higher than those in China, however there are no differences in how imaginative they get to be in their job.  We went one step further as well to look at childhood IC status and current IC status finding that both those with current ICs and past ICs also rate themselves as significantly more imaginative, but again this does not generalize to their jobs.  We have added this into the supplementary material section and readers are directed to the section at the end of the results (line 327-328)  As can be seen below:  *ICs, Imagination and Country Status*  *We then analysed the participant’s reports of their imaginativeness in reference to their IC and country status. We found significantly higher self-report scores of imagination in both those with current t (1,281) = 3.67, p <.001 and past t (1,283) = 3.08, p = .001 ICs. However, this did not remain true for participant report of creativity within their jobs for those with either current t (1,281) = 0.19, p = .426 or past t (1,283) = 0.48, p = .316 ICs.*  *Country differences were found in self-report of imaginativeness, where individuals from the UK reported significantly higher imaginativeness t (1,289) = -2.33, p = .010. Again, when asked how creative one is currently in their job, the difference disappeared t (1,289) = 0.32, p = .376.* |
| 5. The age is only used as a categorical variable, which I think is okay. But I would like to see a reason for how they decided on these categories (e.g., 18-30 and 31-50). When the distribution in two samples (British vs Chinese) are examined in Table 1, we see that about 80% of Chinese sample was between 18 and 30. I would like the researchers to mention this difference and discuss (if relevant) in relation to the results. | This is also a really good idea. We plan to gather age differently in the future and it was not supposed to be gathered in groups, but this was not caught in time to change the method of data collection. The age had been analysed in the supplementary material finding no significant differences, but we still do think this is a limitation to the study and had mentioned in the limitations section, but added this information about the Chinese sample to the limitations.  Below is the limitations addition and the supplementary material section:  *Although there were no differences in age groups (see appendices), it would be useful to be able to look at more fine-grained differences in age, especially because 80% of the Chinese sample was between 18-30.* (line 436)  *Analysis of Age*  *Because there were no specific ages but rather age ranges, we chose to remove the analyses from the main manuscript. We acknowledge this is a limitation to the research. Age groups were analysed finding no age effects χ^2^ (1, N = 181) = 1.06, p = 0.303 without the 50 and over group. The chi square with the over 50 group was also non-significant, but 2 cells were less than 5.* |
| 6. On page 15, it’s stated that “Because ICs can be iICs or POs and both types can have any combination of auditory and visual components for their creators, there are questions around whether there are related differences in face or auditory verbal detection depending on whether one sees or hears their companion. It might be interesting for future research to investigate modality of IC experience and determine whether this relates to top-down processing in only visual tasks, but also auditory.” This question can partially be tested with this dataset, as the researchers are already collecting information about participants’ sensory experiences of their ICs (e.g., Do the participants who report ‘seeing’ their ICs perform differently on the pareidolia task?) | Thank you for bringing this to our attention. The reviewer is right that we can partially answer this question. But given that (a) we can only address part of this question, and (b) we have small groups numbers when we break the ‘IC present’ group into subgroups, we don’t think this analysis is very useful at present. And so, it genuinely is a question for future study. |
| 7. Minor points:  I wonder how the study was advertised and how the recruitment was done – e.g., was the call for participation specify ‘those with ICs’, etc.? | When advertised there was no mention about ICs, but rather a “participants needed for visual tasks and questions about your childhood play” and this would have been the same when translated. We did not want to prime people into thinking about ICs. |
| 8. There are typos throughout the paper that need to be fixed – one major typo being in the running head. | Thank you for this attention to detail. We have amended the running head and have gone through the rest of the manuscript. |
| 9. On Page 11, it’s stated that “Nature of participants’ ICs allowed us to examine associations between performance on the pareidolia task and individual differences in, for example, the ‘sensory’ experiences of an IC. This analysis was exploratory and these associations are reported in Supplementary Materials.”. However, I could not access this file; the supplementary material file only included the SPSS dataset. | We have attached the supplementary materials as this has been reported by the other reviewers as well. Please see reviewer 2 response #12 for more information. |
| 10. There are also some missing information in the references (e.g., for the paper by Majors and Baines (2017), the Journal Title is incorrect). | We’ve gone through and amended 3 citations 1, 8 and 42. We added the correct journal for Majors and Baines (number 42). Hopefully these are the only ones, but they are easily amended if there is another. |

## Supplementary Material

## *Analysis of Interrelations*

### Relations Between Pareidolia and IC perceptual experiences and Imagination

A bivariate correlation was run to determine whether pareidolia hits and false alarms related to the imagination questions and reports of sensory experiences with ICs. Hearing one’s IC was positively related to the amount of pareidolia hits *r =* .280, While other sensory experiences were positively related to pareidolia false alarms *r =* .223 and so was how creative participants rated themselves in their jobs *r* = .146. Pareidolia hits and false alarms were significantly positively related as well *r =* .273. For more inter relations between these variables, see table 1.

## Table 1. Inter relations between variables

| Variable | *n* | 1 | 2 | 3 | 4 | 5 | 6 | 7 |
| --- | --- | --- | --- | --- | --- | --- | --- | --- |
| 1. Can you see your IC | 142 | __ |  |  |  |  |  |  |
| 2. Can you hear your IC | 142 | .472 | __ |  |  |  |  |  |
| 3. Other sensory experiences with IC? | 142 | .344** | .190* | __ |  |  |  |  |
| 4. Total pareidolia hits | 291 | -.019 | .280** | .154 | __ |  |  |  |
| 5.Total pareidolia false alarms | 291 | -.007 | .143 | .223** | .273** | __ |  |  |
| 6.How Creative are you in your job? | 291 | .165* | .005 | .117 | .176 | .146* | __ |  |
| 7.How imaginative are you? | 291 | .195* | .004 | .143 | .099 | .016 | .384** | __ |

**. Correlation is significant at the 0.01 level (2-tailed).

*. Correlation is significant at the 0.05 level (2-tailed).

### Relations Between Pareidolia and IC perceptual experiences Imagination and Culture

We wanted to look at these same variables for each country to account for any differences in associations. First, we looked at only the participants in China. In this group, hearing one’s IC no longer related to the amount of pareidolia hits, but instead other sensory experiences became significant and was associated with pareidolia hits *r =* .261. Other sensory experiences remained a predictor of pareidolia false alarms as well *r =* .291. The two pareidolia scores were also associated with each other *r = .*335. For more inter relations between the variables in the Chinese group see table 2.

Table 2. Interrelations between variables: China

| Variable | *n* | 1 | 2 | 3 | 4 | 5 | 6 | 7 |
| --- | --- | --- | --- | --- | --- | --- | --- | --- |
| 1. Can you see your IC | 77 | __ |  |  |  |  |  |  |
| 2. Can you hear your IC | 77 | -.106 | __ |  |  |  |  |  |
| 3. Other sensory experiences with IC? | 77 | .293** | .148 | __ |  |  |  |  |
| 4. Total pareidolia hits | 185 | .029 | .220 | .261* | __ |  |  |  |
| 5.Total pareidolia false alarms | 185 | -.141 | .163 | .291* | .335** | __ |  |  |
| 6.How Creative are you in your job? | 185 | .208 | .169 | .241* | .118 | .120 | __ |  |
| 7.How imaginative are you? | 185 | .227* | -.064 | -.005 | .042 | .015 | .448** | __ |

**. Correlation is significant at the 0.01 level (2-tailed).

*. Correlation is significant at the 0.05 level (2-tailed).

Next, we looked at the participants in the UK group to determine if there were differences in associations from the combined groups. In the UK, associations with hearing one’s IC was positively related to the amount of pareidolia hits *r = .*373. No other significant associations were found between pareidolia and the perceptual experiences around one’s IC. For more inter relations between the British group see table 3.

## *Table 3. Interrelations between variables: UK*

| Variable | *n* | 1 | 2 | 3 | 4 | 5 | 6 | 7 |
| --- | --- | --- | --- | --- | --- | --- | --- | --- |
| 1. Can you see your IC | 65 | __ |  |  |  |  |  |  |
| 2. Can you hear your IC | 65 | .314* | __ |  |  |  |  |  |
| 3. Other sensory experiences with IC? | 65 | .433** | .234 | __ |  |  |  |  |
| 4. Total pareidolia hits | 106 | -.094 | .373** | -.043 | __ |  |  |  |
| 5.Total pareidolia false alarms | 106 | .187 | .156 | .137 | .156 | __ |  |  |
| 6.How Creative are you in your job? | 106 | .114 | -.132 | -.012 | -.003 | .198* | __ |  |
| 7.How imaginative are you? | 106 | .172 | .060 | .412** | .238* | .096 | .295** | __ |

**. Correlation is significant at the 0.01 level (2-tailed).

*. Correlation is significant at the 0.05 level (2-tailed).

*Analysis of Age*

Because there were no specific ages but rather age ranges, we chose to remove the analyses from the main manuscript. We acknowledge this is a limitation to the research. Age groups were analysed finding no age effects *χ*^2^ (1, *N* = 181) = 1.06, *p* = 0.303 without the 50 and over group. The chi square with the over 50 group was also non-significant, but 2 cells were less than 5.

*ICs, Imagination and Country Status*

We then analysed the participant’s reports of their imaginativeness in reference to their IC and country status. We found significantly higher self-report scores of imagination in both those with current *t* (1,281) = 3.67, *p* <.001 and past *t* (1,283) = 3.08, *p* = .001 ICs. However, this did not remain true for participant report of creativity within their jobs for those with either current *t* (1,281) = 0.19, *p* = .426 or past *t* (1,283) = 0.48, *p* = .316 ICs.

Country differences were found in self-report of imaginativeness, where individuals from the UK reported significantly higher imaginativeness *t* (1,289) = -2.33, *p* = .010. Again, when asked how creative one is currently in their job, the difference disappeared *t* (1,289) = 0.32, *p* = .376.

*Development of Pareidolia Task*

The presence or absence of a face pareidolia in the images presented to participants was established in a pilot study. In this pilot, each image from the final task was presented to a group of seven participants (who did not take part in the full study). Images were presented using Microsoft PowerPoint, in a fixed, random order. Participants were given unlimited time to detect whether or not a face pareidolia was present or absent in each image. All participants detected the face pareidolia in the 24 images that we have classed as “pareidolia present”. None of the participants detected a face pareidolia in the12 images that we have classed as “pareidolia absent”.

*Analyses of Pareidolia Task Performance Using Signal Detection Parameters*

In addition to examining group differences in performance on the pareidolia task using participant’s ‘raw’ scores, we examined group differences using signal detection parameters. We calculated *d'* as a measure of sensitivity. This was found by subtracting the standardized false-alarm rate from the standardized hit rate. We calculated C-score as a measure of response bias. This was found by multiplying the sum of the standardized false-alarm rate and the standardized hit rate by -0.50.

Both C-score and *d'* were not normally distributed. We therefore used non-parametric tests to examine group differences on these outcomes. We tested whether the differences between the IC Groups were significant using a pair of Mann-Whitney U tests. The difference in C-score between the Group without a Childhood IC (median = 0.53, IQR = 0.54) and the Group with a Childhood IC (median = 0.56, IQR = 0.46) was not significant, U = 9822, z = 0.21, p = .83, r = .01, two-tailed test. Similarly, the difference in *d’* (i.e., sensitivity) between the Group without an IC (median = 1.89, IQR = 1.90) and the Group with an IC (median = 1.94, IQR = 1.00) was not significant, U = 8996.50, z = 1.41, p = .16, r = .08, two-tailed test.

The difference in C-score between the Group without a Current IC (median = 0.54, IQR = 0.50) and the Group with a Current IC (median = 0.46, IQR = 0.68) was significant, U = 3446, z = 2.20, p = .028, r = .13, two-tailed test. In contrast, the difference in *d’* between the Group without a Current IC (median = 1.93, IQR = 0.90) and the Group with a Current IC (median = 2.28, IQR = 0.90) was not significant, U = 3132, z = 1.77, p = .08, r = .10, two-tailed test.
