# Supplementary material for: A cross-national study examining imaginary companions and face pareidolia in British and Chinese adults
Source: PLoS One. 2026 Jan 23;21(1):e0325581. doi: 10.1371/journal.pone.0325581 (PMC12829835; doi:10.1371/journal.pone.0325581)
Supplement: Attachment — Submitted filename: Plosone IC Pareidolia_resub02_response.docx [file pone.0325581.s006.docx]

24^th^ November, 2025

Dear Editors,

Thank you for your second review of the manuscript entitled*,* *A Cross-National Study Examining Imaginary Companions and Face Pareidolia in British and Chinese Adults*. We have amended the manuscript according to the feedback and have laid out our changes below. The main text is now 6869 words excluding the abstract and tables/figures. It is now 33 pages in length including the main unblinded text, references, tables and figures. It contains 4 tables 2 figures and appendices.

Sincerely,

Paige E. Davis, PhD, FHEA,

Lecturer in Psychology

University of Leeds

School of Psychology

LS2 9JT

+44 (0)7542 098888

p.e.davis@leeds.ac.uk

**Reviewer #1**

| 1. No response |  |
| --- | --- |

**Reviewer #2**

| 1. I appreciate the authors' through care and attention to my comments and recommended revisions. I have no further recommendations at this time. | We appreciate the time and effort you've put into peer review, thank you. |
| --- | --- |

**Reviewer #3**

| 1. *As I raised a concern in my previous review, I still think that the expected link between hallucinations and ICs (and pareidolia) needs to be rationalized better in the introduction. In the current version of the manuscript, on page 4 the authors state that “…These links between experiencing ICs and individual differences in theory of mind and creativity, as well as similarities in their phenomenology, have resulted in some researchers (e.g., [20, 23]) suggesting that there are important parallels between IC and hallucinations.” I believe the authors could make their reasoning more explicit. | Apologies that this remained unclear. We hope that we have ‘fleshed out’ our reasoning more clearly in the revised manuscript (lines 112-119), where we explain how ICs and hallucinations can be similar in phenomenological terms and may be similar in terms of the factors that play a causal role in their development. |
| --- | --- |
| 2. *Throughout the text, the authors refer to sensory experiences of ICs and how they might be related to hallucinatory experiences. However, as POs are also considered imaginary companions, it would be important to distinguish between invisible ICs and POs, and make separate arguments for their findings when necessary (e.g., visual hallucinations might not be relevant to POs). | We now have written a paragraph in the discussion about this and what future research could investigate (lines 445-452):  Another consideration around iICs and POs is that the experience children are having with them might later result in differences in pareidolia. We theorise that children are using the same type of imagination processing with iICs as with POs. As Taylor and Aguiar argue, children might very well be utilizing their own imagination to embellish the appearance of their PO so that it may look quite different in their mind's eye than in reality (8). Asking participants for details around what their PO looks like to them might be a question to ask adult participants in future research. If it were true that iICs and POs were different imagination processing experiences, the results of many IC studies including this one would need to be reconsidered. |
| 3. *This is also related to my comment in previous version regarding the analysis in terms of sensory experiences. Seeing a PO is not hallucinatory and does not require top-down processing, and given that many adults in the current study report current POs rather than current invisible ICs, I think this should be acknowledged. | We have now acknowledged this in the discussion (lines 442-444).  Parsing apart iICs from POs is especially important with the number of POs in the study sample. Because POs can be seen and may not relate as much to top-down processing, there could be another reason for the results. |
| 4. *On Page 6, it reads “Romagnano and colleagues have even attempted to rule out own-culture and gender biases when participants look at faces”. Here I find it difficult to relate this Romagnano study to the current research question. I believe further information about the Romagnano (2002) study would be helpful. | Apologies that this was not clearer in the previous submission. This was likely due to a typo (‘faces’ versus ‘face pareidolia’). In lines 152-163, we have explained that previous findings re: cultural differences in face processing may not be found when studying face pareidolia because the ‘faces’ being processed by participants are much simpler than ‘real faces’. We note that Romagnano et al. (2022) examined this question and did not find cross-cultural differences in face pareidolia detection, but that their cross-cultural comparison involved groups from northern versus southern Europe. And so there is value in examining other cross-national (i.e., UK versus China) differences in face pareidolia. |
| 5. *I would like to see a more elaborate discussion of the finding that Chinese participants make significantly more false alarms on the task. In discussion section, on page 16, the authors refer to this result and talk about cultural differences in face processing (“It may follow that, with these cultural differences in face processing, there may also translate to differences in pareidolia perception” lines 388-89). I am having a hard time understanding how this result can be explained away with these. Because 1) we do not see cultural differences in hits, 2) the authors stateon page 17, line 394, the authors write “The greater number of false alarms made by Chinese participants may reflect cross-national differences in response biases (49), rather than reflecting differences in cognitive processes, or may simply be statistical ‘noise’.” | Apologies that our previous argument(s) wasn’t clearer. In lines 405-429, we have tried to make our argument more effectively. We note that (in agreement with the reviewer), it seems unlikely that cross-national/cultural difference in face-processing would drive this result. In addition we think it is unlikely that a pareidolia-specific process (i.e., top-down processing) would drive this result.  And so, instead, we propose that it is possible that cross-cultural differences in more generic response biases or in metacognitive confidence may drive this result. However, we note that we know nothing about how generic response biases and/or metacognitive confidence shape performance on the face pareidolia task, so it is hard to be confident that this is a good explanation.  We also expand on the suggestion that the finding may simply be noise/a Type 1 error. We note that the small p-value associated with our finding might be interpreted as this being a weak argument, but that this interpretation is wrong, as a single p-value can’t really be used in that way. |
| 6. *On page 15, the researchers state that “In our research, British adults remembered significantly more ICs in childhood than Chinese adults. One possible explanation for this difference could be that British culture has been traditionally considered to be individualistic, valuing independence, novelty, autonomy and freedom, all values which may lead to creation of an imagined social relationship”. This could very well be true, however, researchers also report that there is no difference in adult creation of ICs and it seems like that their questionnaire taps at remembering of past Ics rather than necessarily of ‘existence of a childhood IC’. Then, I believe that one should be cautious when interpreting results regarding childhood vs. adult IC. When participants report having a childhood IC, we are actually tapping their memory of one (e.g., they might have forgotten that they had one – especially if it’s not talked much in the family), and it might not mean the same thing as when children report having an IC. | We have added this caution and some research that backs up the claim (lines 368-371):  Another possibility is that the questionnaire focused on remembering an IC rather than actually having one. Retrospective report of an IC has been found to be inaccurate in adolescents and misremembering may be a reason to interpret the results around childhood ICs with caution (45). |
| 7&8.  • Gender can be added to the Table 1, to demonstrate basic demographics.  • The SPSS file was available as supplementary material in the previous version, but not in the current version. | - We have added gender to table 1 (lines 638-642) - We were told we could not attach the new SPSS file because it couldn't be opened for the last revision. The corresponding author has now emailed to make certain we can put the updated SPSS file into this revision. Thank you for noting this as it made it possible to contact the editor and query the issue. |
