# Supplementary material for: A cross-national study examining imaginary companions and face pareidolia in British and Chinese adults
Source: PLoS One. 2026 Jan 23;21(1):e0325581. doi: 10.1371/journal.pone.0325581 (PMC12829835; doi:10.1371/journal.pone.0325581)
Supplement: Attachment — Submitted filename: Plosone IC Pareidolia_resub03_response cf.docx [file pone.0325581.s007.docx]

17^th^ December, 2025

Dear Editors,

Thank you for your third review of the manuscript entitled*,* *A Cross-National Study Examining Imaginary Companions and Face Pareidolia in British and Chinese Adults*. We have amended the manuscript according to the feedback you and the reviewer #3 have suggested. We have laid out our changes below. The main text is now 6871 words excluding the abstract and tables/figures. It is now 32 pages in length including the main unblinded text, references, tables and figures. It contains 4 tables 1 figure and appendices.

Sincerely,

Paige E. Davis, PhD, FHEA,

Lecturer in Psychology

University of Leeds

School of Psychology

LS2 9JT

+44 (0)7542 098888

p.e.davis@leeds.ac.uk

**Editor**

| 1. The abstract must be understandable on its own. Please re-write L37-38 as the implication is unclear. It is also unclear what the meaning of “correct face pareidolia” is. | We have re written the sentence to make more sense taking out the word 'correct' as we agree, it could be unclear not having read the study. L37-38 |
| --- | --- |
| L113 – The word ‘typically’ may be changed to ‘sometimes’ | This has now been changed. Thank you. L113 |
| L153 – Images used in all pareidolia tasks do not always resemble faces. Sometimes they are simply noise, in order to elicit false alarms. Please ensure statements such as this are accurate. | These statements are accurate about face pareidolia tasks including the one we used. |
| L368 – This needs to be rewritten. Is the word ‘because’ missing? | We have now changed the sentence to say …which may result in less individual ICs (45). However, with more movement around the globe… L367 |
| L400 – This paragraph begins with a clause related to the previous sentence. Please structure appropriately. | We took out the word 'however' here and started with We think… to structure the sentence more appropriately. L400 |
| L397 – Explain what is meant by “received” | We have changed this word to perceived. L398 |
| L421 – This sentence should be restructured. | This sentence has been broken up into two sentences and made more clear. L420-422. |

**Reviewer #3**

| 1. Typo on Line 169- I think it should read adults' (with apostrophe) | This has now been amended. Thank you for that catch. L169 |
| --- | --- |
| 2) Line 187 - may say 'age and gender data for ...' | We have added this now. Thank you. Ll87 |
| 3) Typo on Line 303 -"in contrast..." ("I" should be capitalized) | The I is now capitalised L303 |
| 4) statistical letters should be italicized in when reporting results (e.g., r, p) | Apologies for this. The statistical letters are now italicised throughout the results section. |
| 5) there is a large overlap between the info provided in Table 4 and Figure 2. They could be integrated or one can be omitted. | We have now cut Figure 2 as it has less information. |
